# Discovery and Development of Caffeic Acid Analogs as Versatile Therapeutic Agents

**DOI:** 10.3390/ph17101403

**Published:** 2024-10-20

**Authors:** Yi Mou, Shuai Wen, Hong-Kai Sha, Yao Zhao, Li-Juan Gui, Yan Wang, Zheng-Yu Jiang

**Affiliations:** 1 College of Pharmacy and Chemistry & Chemical Engineering, Taizhou University, Taizhou 225300, China; wen15298503462@126.com (S.W.); shahongkaitzxy@163.com (H.-K.S.); 15996256893@163.com (Y.Z.); guilijuan12@163.com (L.-J.G.); wangyan20221122@163.com (Y.W.); 2 Jiangsu Key Laboratory of Drug Design and Optimization, China Pharmaceutical University, Nanjing 210009, China

**Keywords:** caffeic acid, caffeic acid derivatives, biological activities, synthesis methods

## Abstract

Caffeic acid (CA) is a polyphenolic acid compound widely distributed in plant seeds. As natural compounds with high research interest, caffeic acid and its derivatives show good activity in the treatment of tumors and inflammation and have antibacterial properties. In recent years, caffeic acid derivatives have been studied extensively, and these derivatives fall roughly into three categories: (1) caffeic acid ester derivatives, (2) caffeic acid amide derivatives, (3) caffeic acid hybrids. These caffeic acid analogues exert mainly antibacterial and antioxidant activities. Among the caffeic acid analogues summarized in this paper, compounds **1g** and CAP10 have good activity against *Candida albicans*, and their MIC_50_ is 32 µg/mL and 13 μM, respectively. In a DPPH assay, compounds **3k**, **5a**, CS2, Phellinsin A and **8j** showed strong antioxidant activity, and their IC_50_ values are 18.6 μM, 67.85 μM, 40.29 μM, 0.29 ± 0.004 mM, 4774.37 ± 137.20 μM, respectively. Overall, compound CAP10 had the best antibacterial activity and compound **3k** had the best antioxidant activity. This paper mainly summarizes and discusses some representative caffeic acid analogs, hoping to provide better drug design strategies for the subsequent development of caffeic acid analogs.

## 1. Introduction

**Figure 1:**
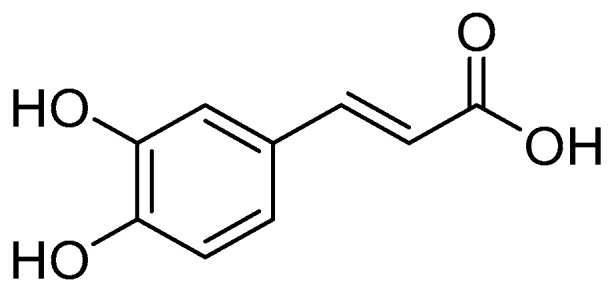
Structure of CA.

Caffeic acid (CA), also known as dihydroxycinnamic acid, is a kind of polyphenolic organic acid [1,2,3,4,5,6] (Figure 1). It is widely found in plant seeds, specifically in coffee, vegetables, fruits, olive oil, grains and other Chinese medicinal materials [7,8,9,10,11,12,13]. Vegetables and fruits rich in caffeic acid in nature mainly include spinach, thyme, apples, pears, blueberries and so on. Caffeic acid is also found in many Chinese herbs that are widely used worldwide, such as dandelion, cinnamon, salvia miltiorrhiza, hawthorn, honeysuckle and eucommia ulmoides, as well as plants in the umbrella family and honeysuckle family [2]. In pharmacopoeia, the quantitative analysis of caffeic acid is an important quality control index to ensure that the caffeic acid content in medicinal materials meets the prescribed standards. For example, in the Chinese Pharmacopoeia (2020 edition), the items related to the identification and content determination of dandelion have changed: the caffeic acid content is no longer determined, but is replaced by chicory acid as a reference product. At present, the commonly used methods for caffeic acid quantitative analysis mainly include high performance liquid chromatography and volumetric titration [14,15]. The structural formula for caffeic acid has two phenolic hydroxyl groups [16,17,18]. These two phenolic hydroxyl groups are good donors of hydrogen atoms and are able to react with free radicals, thus neutralizing their activity. This gives caffeic acid the ability to scavenge peroxyl radicals and hydroxyl radicals, hence it exhibits some antioxidant activity [19,20,21]. Meanwhile, in the structure of caffeic acid, the conjugation between the double bond and the benzene ring stabilizes the two phenolic hydroxyl groups as well as the electron transfer process in the reaction of free radicals, which further improves the antioxidant effects [22]. As a natural product with wide application prospects, research on caffeic acid and its homologues has become a hot topic, and some caffeic acid analogues have good antibacterial and antioxidant effects. Therefore, the authors believe that it is necessary to carry out a review of caffeic acid analogues, which can provide better drug design strategies for subsequent caffeic acid analogues research. At the same time, this review is expected to provide some data to support the subsequent development of better caffeic acid analogues [23,24,25,26].

## 2. Caffeic Acid Derivatives

Caffeic acid derivatives have profound prospects for research due to their high biological activity and wide-ranging biological sources [27,28,29,30]. The synthesis of caffeic acid derivatives has been worked on extensively, and these derivatives fall roughly into three categories: (1) caffeic acid ester derivatives, (2) caffeic acid amide derivatives, (3) caffeic acid hybrids. Caffeic acid ester derivatives mainly include phenethyl caffeate, caffeoylquinic acid, compounds **1g** and CAP10. Among them, compounds **1g** and CAP10 have good antibacterial activity. Caffeic acid amide derivatives mainly include compounds **3k**, **4′d** and **5a**. Studies have shown that these compounds have a certain antioxidant capacity, and compounds **3k** and **5a** in particular have a strong antioxidant capacity. In addition, the caffeic acid hybrids reported mainly contain compounds CS2, Phellinsin A and **8j**. The DPPH assay indicated that these compounds also have a good antioxidant capacity (Table 1).

### 2.1. Caffeic Acid Ester Derivatives

Among caffeic acid derivatives, the synthesis and bioactivity of ester derivatives are studied most widely [42,43,44,45,46]. These derivatives are mainly synthesized through a reaction between caffeic acid and different alcohols [47,48,49,50]. Thus, the main differences in the structure of caffeic ester derivatives result from the differences in alcohol. Alcohols used in these reactions include open chain alkyl alcohols, aromatic alcohols and heterocyclic alcohols.

**Figure 2:**
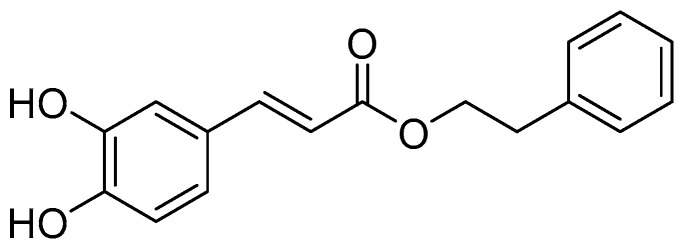
Structure of CAPE.

Phenethyl caffeate (CAPE) (Figure 2) is an extensively studied ester derivative [51,52,53,54]. It was found that phenethyl caffeic acid shows a good activity in the treatment of tumors and inflammation and has antibacterial properties [55,56,57,58,59,60,61,62]. It has been confirmed that phenethyl caffeate shows a good inhibitory activity against *S. mutans*, and its MIC_50_ is 5.2 ± 0.8 µg/mL [31]. Considering the bioactivities of CAPE, numerous derivatives of CAPE have been developed. But the large-scale preparation of CAPE is a hot and difficult research topic. Synthetic methods for the preparation of CAPE can be categorized as chemical synthesis and biosynthesis. Chemical synthesis methods (Table 2) for CAPE mainly include the direct catalysis method [63,64,65], the halogen-substituted hydrocarbon method [63], the acyl chloride method [66], the witting reaction [67,68], the malonic acid monoester method [69] and the one pot method [70]. Each of these synthesis methods has its own advantages and disadvantages. For example, catalytic esterification has simple reaction conditions but is costly and time consuming; the halogenated hydrocarbons method is mild but costly and cumbersome; the witting reaction conditions are not harsh, and the yield is considerable, but the triphenylphosphine used is expensive, and it is easy to pollute the environment; the one-pot synthesis method has low cost and high yield but requires the use of more toxic piperidine and pyridine.

Two phenolic hydroxyl groups are important groups in CAPE, and SAR studies have shown that substitution of one of the hydroxyl groups can enhance its activity. In particular, substituting the benzene ring with some electron-withdrawing groups can increase the activity [71].

**Figure 3:**
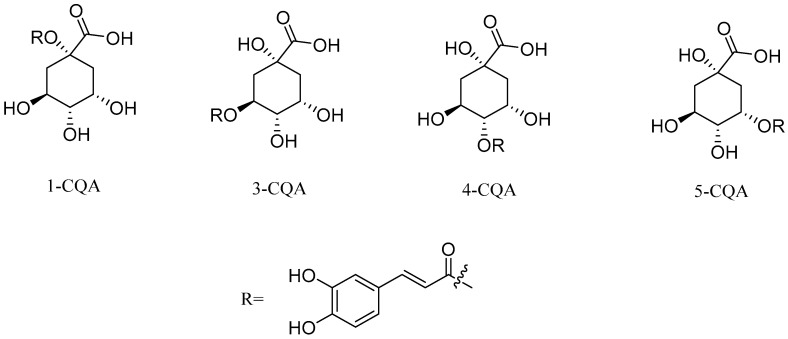
The structural formulas of MCQA.

**Figure 4:**
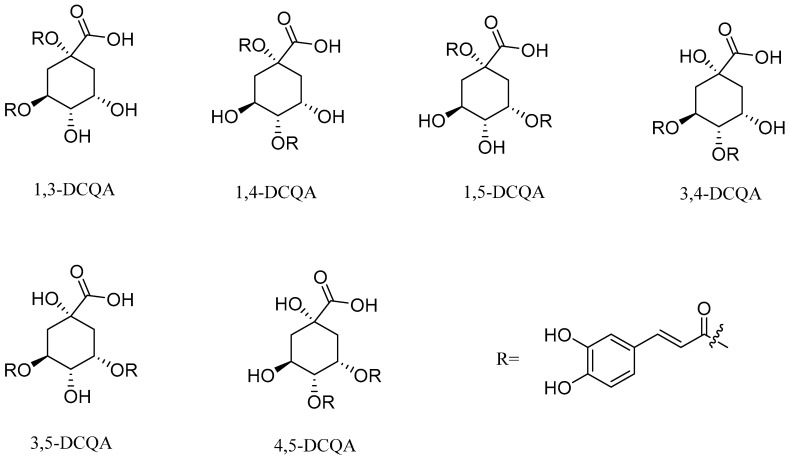
The structural formulas of DCQA.

**Figure 5:**
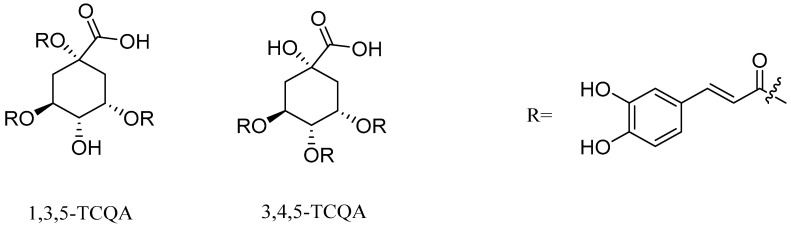
The structural formulas of TCQA.

Similar to CAPE, caffeoylquinic acid is a widely studied ester derivative [72,73,74]. Caffeoylquinic acid belongs to organic acids containing phenolic rings, which are widely distributed in nature, such as Chinese herbs, fruits and so on [75,76]. Fruits rich in caffeoylquinic acid in nature mainly include apples and cherries. Among the Chinese herbs widely used around the world, there are also some rich in caffeoylquinic acid, such as honeysuckle, eucommia ulmoides, cocklebur, hawthorn, tuberous root and so on. The quantitative analysis of caffeoyl quinic acid in pharmacopoeia is mainly carried out using high performance liquid chromatography. This method is widely used for the determination of caffeoyl quinic acid compounds with a high accuracy and precision. Specific examples include the HPLC-PDA method for the determination of the caffeoylquinic acid component in *Azolla imbricata* [77,78]. The results show that the method has a good repeatability and reliability. Studies have shown that caffeoylquinic acid has anti-tumor, antioxidant, anti-inflammatory, cardiovascular protection, neuroprotection and other biological activities [79,80,81,82]. Caffeoylquinic acid is esterified from caffeic acid and quinic acid. This series of compounds mainly consists of monocaffeoylquinicacids (MCQA, Figure 3), dicaffeoylquinicacids (DCQA, Figure 4) and tricaffeoylquinicacids (TCQA, Figure 5) [83]. Caffeoyl quinic acid compounds have great potential for the treatment of some diseases. For example, 3-CQA isolated from honeysuckle has significant antibacterial effects against *Staphylococcus aureus* and *Escherichia coli* [84]. Farias-Pereira et al. [85] conducted a screening test on extracts of green coffee beans and 5-CQA through an obesity model and found 5-CQA to be the main weight loss component of coffee beans. In addition, it has been reported that 5-CQA has good inhibitory activity against *Stenotrophomonas maltophilia*, and its MIC_50_ is in the range of 8 to 16 µg/mL [33]. Han et al. [86] compared the activities of three kinds of dicaffeinoquinic acid and found that 3, 4-DCQA, 3, 5-DCQA and 4, 5-DCQA all had significant antibacterial effects. In addition, 3, 5-DCQA can promote the mRNA expression of phosphoglycerate kinase 1 in human neuroblastoma cells and increase the level of intracellular ATP, thus exerting neuroprotective effects on neurons [87].

In light of the bioactivities of caffeoylquinic acid, numerous derivatives of caffeoylquinic acid have been developed. These compounds are composed of many isomers and are prone to isomerization. The activity of caffeoylquinic acid is closely related to the absolute configuration [79]. Therefore, the study of optic isomers could be studied in future studies.

**Figure 6:**
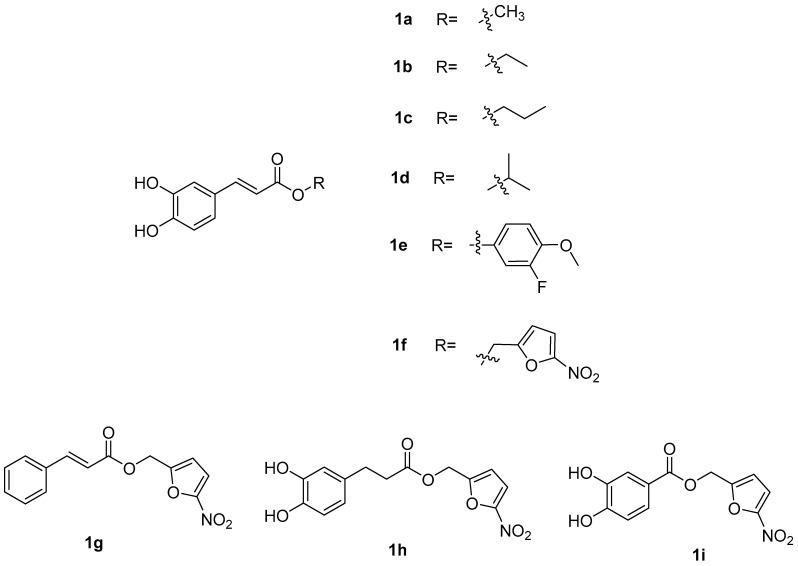
Structures of caffeic acid ester derivatives.

De Vita et al. [32] used different alcohols (fatty alcohols, aromatic alcohols) to synthesize different caffeic ester derivatives through an esterification reaction (Figure 6). The study evaluated the effects of these derivatives on the formation and destruction of *Candida albicans* biofilm, showing that caffeic acid was more active than fluconazole on mature biofilms after esterification. In particular, compounds **1f**, **1g** and **1i** showed higher activity against mature biofilms than fluconazole, and their MIC_50_ values were 128, 64 and 64 µg/mL, respectively. In addition, the activity of these three compounds on biofilm formation was higher than that of fluconazole.

**Figure 7:**
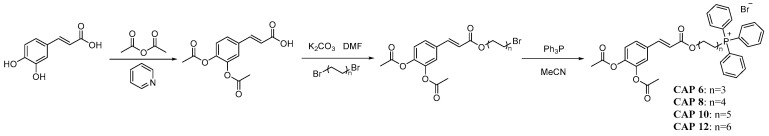
Structures of CAPs.

Lukac et al. [34] synthesized several phosphor derivatives (CAPs) with different carbon chain lengths using caffeic acid as a starting material (Figure 7). Their biological activity evaluation showed that CAPs exhibited significantly stronger cytotoxic activity in comparison to CA. The results of an antimicrobial test confirmed that CAPs have significant activity compared with caffeic acid, and their MIC_50_ values for *Gram-positive bacteria* and *Candida albicans* ranged from 13 μM to 57 μM. These novel compounds appeared to be promising antimicrobial agents for further research.

### 2.2. Caffeic Acid Amides Derivatives

Caffeic acid amide derivatives, serving as important derivatives of caffeic acid, are widely found in natural plants and have a high biological activity [88]. Caffeic acid amide derivatives can protect endothelial cells from oxidation [89]. These derivatives are mainly synthesized from caffeic acid with different amines, and the amide groups in the structure have high stability. Therefore, in recent years, researchers have conducted more in-depth research on these kinds of derivatives.

**Figure 8:**
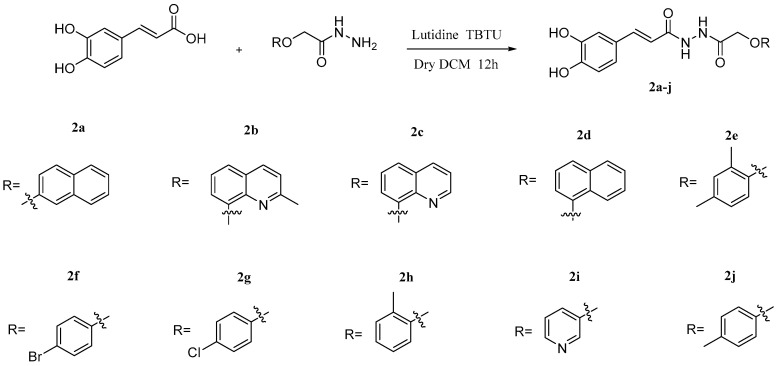
Structures of caffeic acid amides derivatives **2a–j**.

Al-Ostoot et al. [90] synthesized a series of caffeic acid derivatives **2a–j** via etherification and coupling action (Figure 8), and their anti-inflammatory and analgesic effects were tested. The results showed that most of the caffeic acid derivatives exerted comparable activity to the reference compound celecoxib. Among these derivatives, compounds **2f** and **2g** have better activity. And the Cyclooxygenase-I (COX-I)/Cyclooxygenase-II (COX-II) activity ratio of **2f** and **2g** suggested that these two compounds have the same inhibitory effect. The US Environmental Protection Agency has signed off on a rule banning most uses of dichloromethane in an effort to protect public health. Long-term exposure to dichloromethane can cause diseases such as cancer and have adverse effects on the human body and the environment. Dichloromethane was used in the synthesis of the target compound, and it is suggested that the authors avoid using dichloromethane in the subsequent synthesis of such compounds, and use other solvents instead, such as DMF.

**Figure 9:**
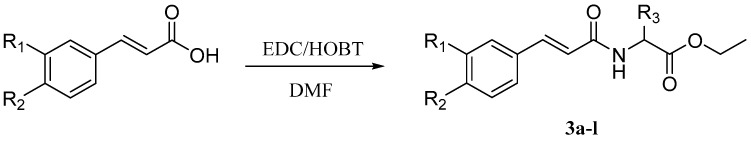
Synthesis method for CAES.

Wang et al. [35] synthesized twelve N-hydroxycinnamoyl amino acid amide ethyl esters (CAES, Figure 9, Table 3) by using amino acids and caffeic acid homologs as the initial material. Their results showed that all CAES have the ability to scavenge free radicals, and N-caffeoyl amide derivatives showed higher radical scavenging activity than N-feruloyl amide derivatives. Among the series of derivatives, compound **3k** has the strongest free radical scavenging ability and its IC_50_ value is 18.6 µM.

**Figure 10:**
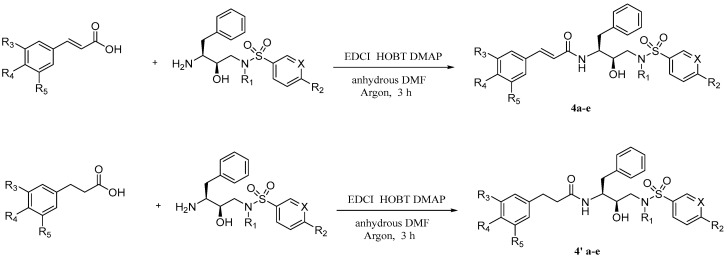
The synthesis method for **4a–e** and **4′a–e**.

Considering the special properties (flexible chain, small steric resistance) of caffeic acid and its homologue, Zhu et al. [36] prepared several derivatives that can be used for the dual inhibition of HIV-1 protease (PR)/reverse transcriptase (RT) (Figure 10, Table 4). These inhibitors were synthesized by reacting substituted cinnamic acids or substituted phenylpropionic acids with corresponding amines in the presence of 1-ethyl-3-(3-dimethylaminopropyl)carbodiimide (EDCI)/1-hydroxybenzotriazole (HOBT)/4-dimethylaminopyridine (DMAP) at 0–25 °C for 2–3 h. Among the series of derivatives, the anti-PR activity of compound **4′d** was 19 times higher compared with the control DRV, and its IC_50_ value is 0.081 nM. Compound **4′c** exhibited an excellent anti-RT IC_50_ value of 0.43 µM.

**Figure 11:**
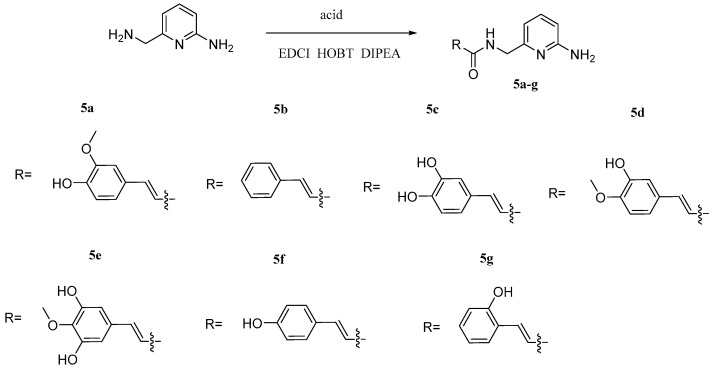
The synthesis method for **5a–g**.

Li et al. [37] synthesized a novel class of proteolytic enzyme β-secretase (BACE1) inhibitors with free radical-scavenging activity (Figure 11). These inhibitors were synthesized via molecular hybridization by using 6-(aminomethyl)pyridin-2-amine and corresponding substituted acids as raw materials under the conditions of EDCI/HOBT. Activity test showed that compound **5a** has strong inhibitory and antioxidant activity against BACE1 and DPPH, indicating that compound **5a** has good research value for follow-up research.

### 2.3. Caffeic Acid Hybrids

In recent years, more and more cases have been reported using hybrid strategies to combine caffeic acid with clinical drugs to obtain new molecular entities with good activity. The advantage of these hybrids is that they can simultaneously maintain the pharmacological activity of caffeic acid homologues and clinical drugs, while enhancing synergies [91].

**Figure 12:**
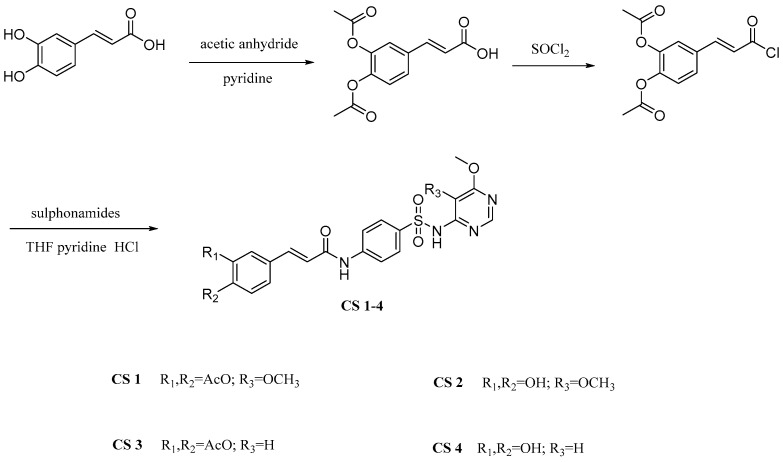
The synthesis method for CSs.

Peng et al. [38] prepared several hybrids (CSs) by linking caffeic acid to sulfonamides using a coupling strategy (Figure 12). These hybrids were evaluated for a series of biological activities and the results indicated that CSs have good effects in a range of antioxidant, anticoagulant and antibacterial activities, present cytotoxicity and promote chondrocyte proliferation.

**Figure 13:**
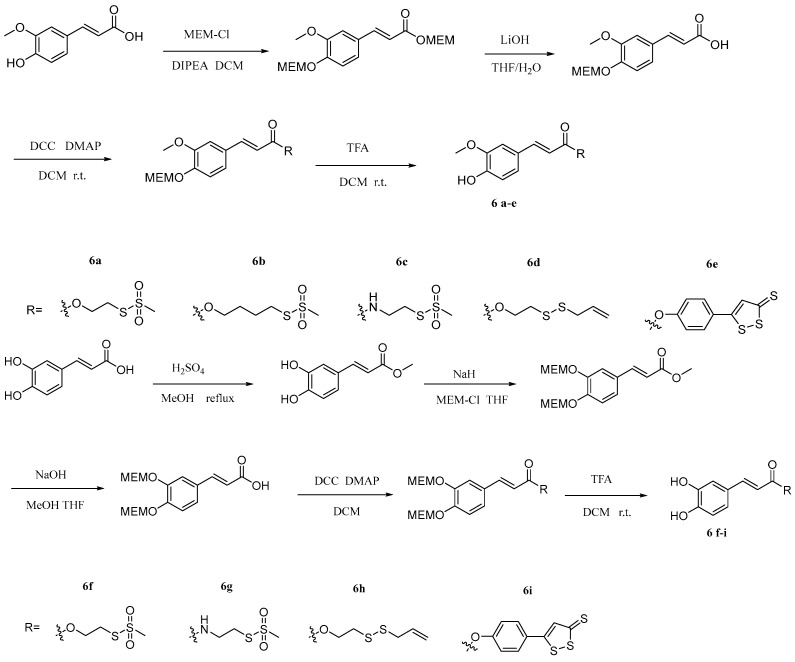
The synthesis method for **6a–i**.

Gabriele et al. [39] synthesized a set of new sulfurated drug hybrids of cinnamic acids (Figure 13) and tested their inhibition of STAT3 and NF-κB transcription factors. The results showed that most of these compounds can bind to STAT3 selectively. In addition, some drugs can inhibit HCT-116 cell proliferation and NF-κB transcriptional activity, and the corresponding IC_50_ values are in the micromolar range. These hybrids have great significance for the subsequent development of multi-target anticancer drugs. The use of dichloromethane in the synthesis of the target compound can cause adverse effects on humans and the environment and is not in compliance with the U.S. Environmental Protection Agency’s regulations restricting the use of dichloromethane. It is suggested that the authors should avoid the use of dichloromethane in the subsequent synthesis of similar compounds, and use other solvents instead, such as acetonitrile.

**Figure 14:**
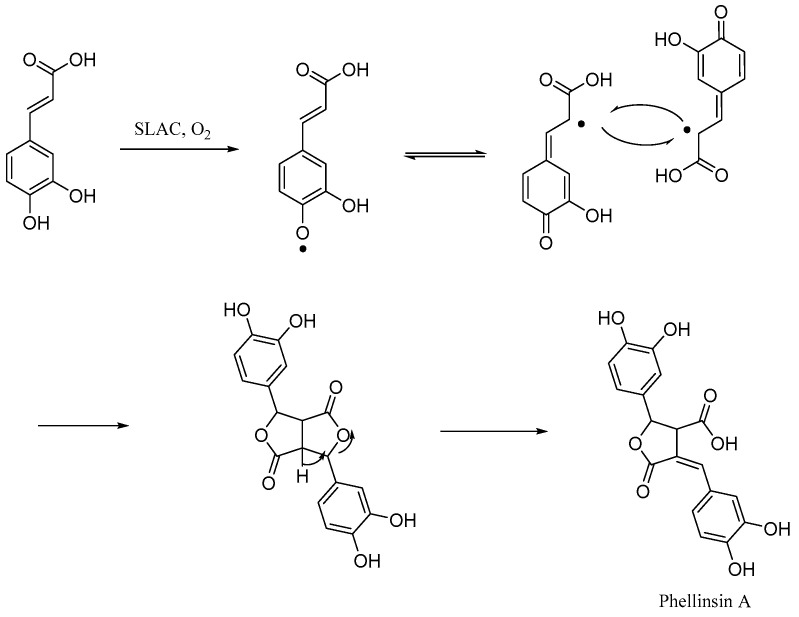
The synthesis method for Phellinsin A.

Nemadziva et al. [40] synthesized a dimer compound (Phellinsin A). The results showed that Phellinsin A (Figure 14) had a strong DPPH free radical scavenging ability and equivalent antioxidant capacity (TEAC). Compared to caffeic acid, these two capabilities increased by 1.5 times and 1.8 times, respectively. Furthermore, in aqueous media and an acidic pH, Phellinsin A exhibited improved solubility properties and good stability.

**Figure 15:**
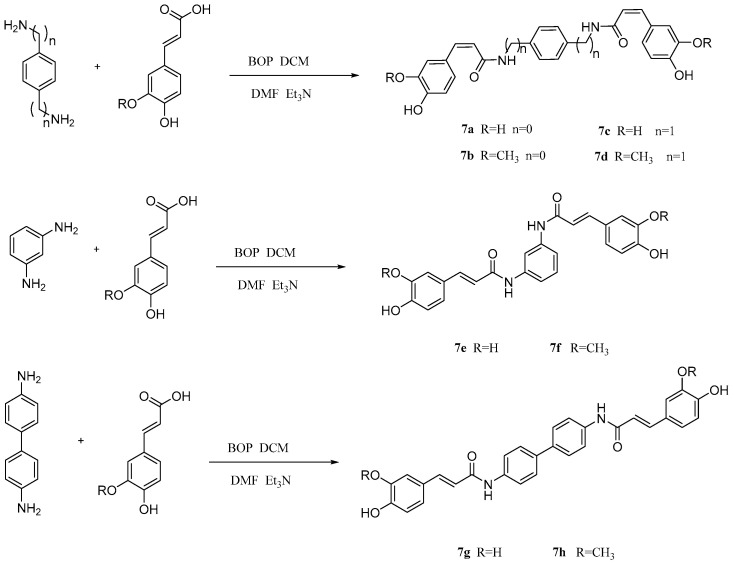
The synthesis method for **7a–h**.

In addition, He et al. [92] prepared several kinds of novel dimers using ferulic acid and CA (Figure 15), then tested the therapeutic effect of these dimers on Alzheimer’s disease (AD). It turned out that compound **7h** had a strong protective function on HT22 cells of mice hippocampal neurons, and no obvious cytotoxicity. These data suggest that compound **7h** is an important reference for the development of multifunctional drugs in the AD class.

**Figure 16:**
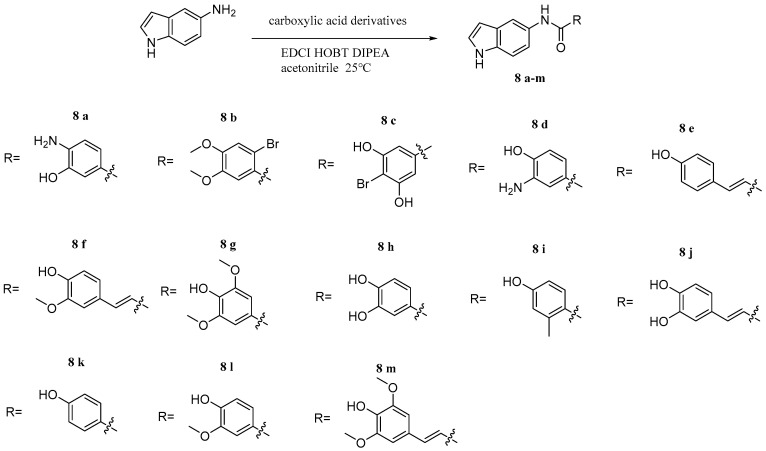
The chemical structures of **8a–m**.

Elkamhawy et al. [41] synthesized some hybrids based on indole groups and caffeic acid homologues (Figure 16). The antioxidant activity of synthesized compounds was evaluated by radical scavenging assays. The DPPH assay demonstrated that these hybrids are more active free radical scavenging agents. In particular, compound **8j** presented the highest antioxidant activity with a ferric reducing ability of plasma (FRAP) assay value of 4774.37 ± 137.20 µM Trolox eq/mM sample. Taken together, compound **8j** was shown to be optimized to maximize its antioxidant capacity.

## 3. Conclusions

The design and evaluation of the therapeutic activity of caffeic acid derivatives have attracted more and more attention, and it turns out that some caffeic acid analogues have good antibacterial and antioxidant effects. Among the caffeic acid derivatives summarized in this paper, the caffeic acid ester derivative compound CAP10 has the best antibacterial activity, while the caffeic acid amide derivative compound **3k** has the best antioxidant activity. The structure–activity relationship of caffeic acid analogues has been preliminarily understood. Biological activity of these compounds is closely related to the hydroxyl group on the benzene ring. Specifically, with some electron-absorbing substituent groups on the benzene ring, the derivatives have stronger biological activity. Therefore, new caffeic acid derivatives can be designed by changing the substituents on the benzene ring.

Caffeic acid and its derivatives have been applied for treating cardiovascular and cerebrovascular diseases. For example, caffeic acid tablets can be used to treat leukopenia and sodium ferulate can be used to treat atherosclerosis [93,94]. In addition, the exploration of new applications is also becoming a new research hotspot. For instance, caffeic acid analogues can be used as additives for food preservation due to their antioxidant activity. All in all, caffeic acid analogues are natural active substances with good application prospect. However, hurdles remain in this field. For example, further research on targeting caffeic acid and caffeic acid hybrids is needed. It is believed that with the continuous deepening of research, more and more caffeic acid derivatives with better therapeutic effects will be developed.

## Figures and Tables

**Table 1:** List of chemical structures and therapeutic activities of representative caffeic acid derivatives.

Types of Derivatives	Chemical Structures	Biological Activities	References
CA-ester derivatives	CA-phenethyl ester 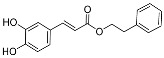	Showed antibacterial activity MIC_50_ 5.2 ± 0.8 µg/mL for *S. mutans*	[31]
	Compound **1g** 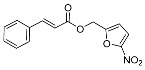	Showed antibacterial activity MIC_50_ 32 µg/mL for *Candida albicans*	[32]
	5-CQA 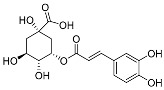	Showed antibacterial activity MIC_50_ 8–16 µg/mL for *Stenotrophomonas maltophilia*	[33]
	Compound CAP10 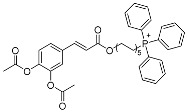	Showed antibacterial activity MIC_50_ 13 μM for *Candida albicans*	[34]
CA-amides derivatives	Compound **3k** 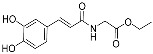	Showed antioxidant activity IC_50_ 18.6 μM	[35]
	Compound **4′d** 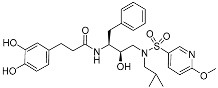	Showed antiviral activity IC_50_ 0.081 nM for HIV-1 protease	[36]
	Compound **5a** 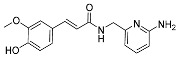	Showed antioxidant activity IC_50_ 67.85 μM	[37]
CA-hybrids	Compound CS2 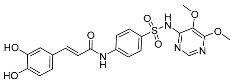	Showed antioxidant activity IC_50_ 40.29 μM	[38]
	Compound **6i** 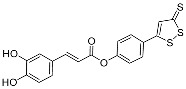	Showed antitumor activity IC_50_ 46.7 μM for HCT-116 cells	[39]
	Compound Phellinsin A 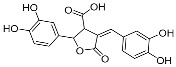	Showed antioxidant activity IC_50_ 0.29 mM	[40]
	Compound **8j** 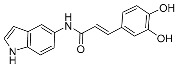	Showed antioxidant activity IC_50_ 4774.37 μM	[41]

**Table 2:** The chemical synthesis methods for CAPE.

Synthetic Methods	Reagents	Solvent	Reaction Temperature	References
Direct Catalysis Method	Caffeic acid, phenethyl alcohol, toluene-p-sulfonic acid	Benzene	Reflux	[64]
Direct Catalysis Method	Caffeic acid, phenethyl alcohol, dicyclohexylcarbodiimide and dimethylaminopyridine	THF/CH_2_Cl_2_ (1:1)	Room temperature	[65]
Halogen-Substituted Hydrocarbon Method	Caffeic acid, β-phenyl ethyl bromide, sodium hydroxide	Hexamethylphosphoramide (HMPA)	Room temperature	[63]
Acyl Chloride Method	Caffeic acid, phenethyl alcohol, SOCl_2_, pyridine	Nitrobenzene	Refluxing temperature	[66]
Witting Reaction	3,4-Dihydroxy benzaldehyde, triphenylphosphonic acid phenylethanol ester chloride, potassium carbonate	CHCl_3_/dioxane (1:1)	Room temperature	[67,68]
Malonic Acid Monoester Method	Malonic acid, phenethyl alcohol, DPAT, 3,4-dihydroxy benzaldehyde	Toluene	80 °C	[69]
One Pot Method	Isopropylidene malonate, phenethyl alcohol, 3,4-dihydroxy benzaldehyde, pyridine, piperidine	Toluene	60 °C–room temperature	[70]

**Table 3:** The structures of **3a–l**.

Compound	R_1_	R_2_	R_3_
**3a**	OCH_3_	OH	CH_3_
**3b**	OCH_3_	OH	CH(CH_3_)_2_
**3c**	OCH_3_	OH	CH_2_CH(CH_3_)_2_
**3d**	OCH_3_	OOCCH_3_	CH_3_
**3e**	OCH_3_	OOCCH_3_	CH(CH_3_)_2_
**3f**	OCH_3_	OOCCH_3_	CH_2_CH(CH_3_)_2_
**3g**	OH	OH	CH_3_
**3h**	OH	OH	CH(CH_3_)_2_
**3i**	OH	OH	CH_2_CH(CH_3_)_2_
**3j**	OH	OH	CH(CH_3_)CH_2_CH_3_
**3k**	OH	OH	H
**3l**	OH	OH	CH_2_C_6_H_6_

**Table 4:** The structures for **4a-e** and **4′a–e**.

Compound	R_1_	R_2_	R_3_	R_4_	R_5_	X
**4a**	CH_2_CH(CH_3_)_2_	OCH_3_	OH	OH	H	CH
**4b**	CH_2_CH(CH_3_)_2_	NH_2_	OH	OH	H	CH
**4c**	CH_2_CH(CH_3_)_2_	SCH_3_	OH	OH	H	CH
**4d**	CH_2_CH(CH_3_)_2_	OCH_3_	OH	OH	H	N
**4e**	CH_2_CH_2_CH_3_	OCH_3_	OH	OH	H	CH
**4′a**	CH_2_CH(CH_3_)_2_	OCH_3_	OH	OH	H	CH
**4′b**	CH_2_CH(CH_3_)_2_	NH_2_	OH	OH	H	CH
**4′c**	CH_2_CH(CH_3_)_2_	SCH_3_	OH	OH	H	CH
**4′d**	CH_2_CH(CH_3_)_2_	OCH_3_	OH	OH	H	N
**4′e**	CH_2_CH_2_CH_3_	OCH_3_	OH	OH	H	CH
